# From the archives: *Ear apical degeneration 1* is essential for maize ear development, SHOOT MERISTEMLESS regulates floral fate, and the role of profilin in pollen tube growth

**DOI:** 10.1093/plcell/koad080

**Published:** 2023-03-17

**Authors:** Jessica Franco

**Affiliations:** Assistant Features Editor, The Plant Cell, American Society of Plant Biologists, USA; Department of Plant Pathology, Washington State University, Pullman, WA, USA

## June 2022: *Ear apical degeneration 1* is essential for maize ear development

The demand for staple food crops such as maize continues to increase as global population growth soars. Maize ear length (EL) is proportional to grain yield (Li et al. 2018). Thus, genetic information regulating EL can inform strategies to maximize grain yield. **Pei et al. (2022)** characterized the maize shortened ear mutant, *ear apical degeneration 1* (*ead1*), using microscopy and anatomical analyses. The authors found that the *ead1* short ear phenotype was due to programmed cell death at the apex of the female inflorescence during development. The *EAD1* locus was identified using map-based cloning analyses. EAD1 is an aluminum (Al)–activated malate transporter (AMLT) which are ion channels central to various physiological processes. Strikingly, malate concentrations in the middle and apical parts of immature ears were significantly reduced in *ead1* compared to wild type, suggesting that *EAD1* plays a role in malate transport in these tissues. The authors functionally validated EAD1 as a malate efflux transporter using the 2-electrode voltage clamp technique coupled with stable isotope labeling. Furthermore, exogenous malate injection into *ead1* immature ears rescued the short ear phenotype. This work provided a stepping stone towards improved grain yields by targeting EAD1.

**Figure.**
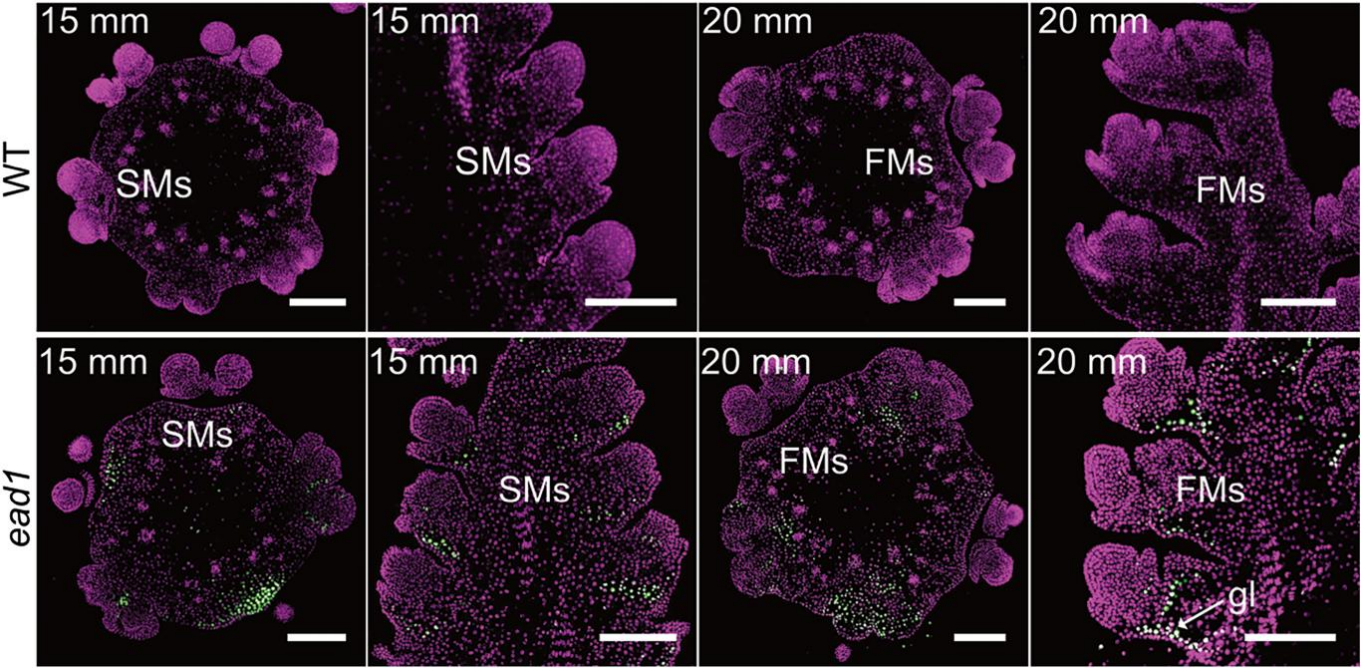
PCD in *ead1* apical ears. TUNEL assay to detect PCD in apical ears in WT and *ead1*. Transverse (column 1 and 3) and longitudinal (column 2 and 4) sections, counterstained with propidium iodide. Signal indicative of PCD was detected in *ead1* ears, but not in the WT. gl, glume; SMs, spikelet meristems; FMs, floral meristems. Scale bars = 200 μm. Adapted from Pei et al. (2022) Figure 2.

## June 2018: SHOOT MERISTEMLESS regulates floral fate

The KNOXI transcription factor *SHOOT MERISTEMLESS* (*STM*) is involved in maintaining the shoot apical meristem. *STM* is not expressed in the floral primordial; however, *STM* expression resumes during the production of flower organs (Long et al. 1996). The loss of *STM* function is lethal at the seedling stage providing challenges to study its role during flower development. To overcome this obstacle, **Roth et al. (2018)** generated artificial microRNAs driven by the *APETALA* (*AP1*) promoter to silence *STM* expression at different developmental stages. *AP1* is a transcription factor that orchestrates flower meristem identity (Bowman et al. 1993). Silencing *STM* in an AP1 loss-of-function mutant exacerbated the leafy-flower phenotype, suggesting that *STM* functions synergistically with *AP1* to regulate flower meristem identity. The authors performed RNA-sequencing analysis to understand the impact of *STM* expression prior to anthesis. Multiple genes involved in floral transition, fate, and patterning were activated upon *STM* induction. The upregulation of *UNUSUAL FLORAL ORGANS* (*UFO*) was the most significant. Genetic analysis revealed that *STM* and *UFO* interact in an *AP1*-dependent manner to modulate flower meristem identity. This work demonstrated that *STM* has distinct role at different developmental stages and meristem tissues.

## The role of profilin in pollen tube growth

The control of pollen germination and pollen tube growth depends on a complex interplay between the actin cytoskeleton and signaling cascades. Profilin, a small actin-binding protein, is abundant in pollen tubes and modulates the actin cytoskeleton (Cai et al. 1997). At the time of this study, it was known that profilin is phosphorylated in animal cells, but the function of phosphorylated profilin was unknown. The role of profilin in plant cell signaling was largely unexplored. **Clarke et al. (1998)** investigated the function of profilin during pollen tube growth. The authors first purified native profilin from poppy pollen, which was fully functional in vitro and in living cells. Next, the authors examined the in vitro phosphorylation profile of poppy pollen extracts after treatment with excess native poppy profilin. The phosphoproteins of the pollen cytosol, microsomes, and a combination of cytosol and microsomes were detected using autoradiography. After profilin treatment, pollen cytosolic proteins exhibited significant decreases in phosphorylation in a concentration-dependent manner. To test the specificity of profilin treatment, the authors treated poppy extracts with poppy profilin and 2 maize profilins. The phosphoprotein profiles were similar, but there were distinct qualitative differences between maize and poppy profilin treatments. These results suggested that profilin interactions with target proteins are concentration and species dependent. Overall, this study provided foundational knowledge that profilins are involved in signaling pathways within plant cells.
